# Crosstalk Between Astrocytes and Microglia: An Overview

**DOI:** 10.3389/fimmu.2020.01416

**Published:** 2020-07-16

**Authors:** Agata Matejuk, Richard M. Ransohoff

**Affiliations:** ^1^Department of Immunology, Collegium Medicum, University of Zielona Góra, Zielona Góra, Poland; ^2^Third Rock Ventures, Boston, MA, United States; ^3^Department of Cell Biology, Harvard Medical School, Boston, MA, United States

**Keywords:** microglia, astrocytes, neurons, glia, CNS, neurological disorders, neurodegeneration

## Abstract

Based on discoveries enabled by new technologies and analysis using novel computational tools, neuroscience can be re-conceived in terms of information exchange in dense networks of intercellular connections rather than in the context of individual populations, such as glia or neurons. Cross-talk between neurons and microglia or astrocytes has been addressed, however, the manner in which non-neuronal cells communicate and interact remains less well-understood. We review this intriguing crosstalk among CNS cells, focusing on astrocytes and microglia and how it contributes to brain development and neurodegenerative diseases. The goal of studying these intercellular communications is to promote our ability to combat incurable neurological disorders.

## Introduction

Every organ possesses one cell type whose properties incarnate and define its function. For the central nervous system (CNS), that cell is the neuron. Synaptic communication among neurons is organized in neural circuits, which carry out humanity-defining tasks such as written language, as well as brain function, ranging from breathing to motor behavior to perception. Each of the populations of non-neuronal cells of the adult CNS are remarkably adapted to support neuronal function: astrocytes maintain ionic and neurotransmitter homeostasis, refine synaptic connections, and provide neuronal metabolic substrates; microglia monitor synaptic elements and networks, responding to dyshomeostasis by inducing or removing synaptic elements and by modulating neuronal activity; oligodendrocytes elaborate myelin sheaths, which protect and nourish myelinated neuritic segments. Microglia and astrocytes respond to neuronal injury with programs that include proliferation, morphological alterations, mediator production, and engulfment of cells and subcellular elements. These changes represent the CNS tissue response to damage or degeneration.

During development and early-adult life, forces crafted by evolution optimize the CNS structure and function for reproductive fitness and survival. Given that human life-span now extends well-beyond the end of reproductive capacity, it's axiomatic that, while CNS disorders of aging evoke a tissue response, that reaction isn't shaped by evolution to respond specifically to the challenges posed either by aging or by the ongoing pathogenic process. Research into neuroinflammatory glial biology involves characterizing this tissue response and defining its effects on the outcomes of neurological disorders, as well as searching for therapies to ameliorate injurious glial reactions and restore homeostasis.

As noted above, astrocytes and microglia exert their primary functions toward neurons, and much research addresses the dyadic interactions: microglia-neuron and astrocyte-neuron. It is also timely to consider how microglia and astrocytes signal to each other, to obtain a more-comprehensive account how their behavior is regulated in the complex context of CNS injury or disease. This review takes the approach of introducing briefly each cell type in relation to its interactions with neurons, followed by a series of “embryonic” illustrating how microglia and astrocytes can communicate. Finally, these interactions will be placed in the setting of varied CNS disorders. In each circumstance, the relevant outcome of astrocyte-microglial communication will be health of the individual neuron or integrity of the neural circuit.

## Microglia

More than 100 years ago, in 1919 Pio del Rio-Hortega published an article in which he introduced the term “microglia.” He used an improved silver-staining method to delineate microglia and to discriminate them from oligodendrocytes, the other cellular component of the Third Element of the CNS (1). Today we can enjoy the observation of microglia in 3D at nanometer-resolution visualized by volumetric ultrastructural reconstruction using serial block face scanning electron microscopy (SBEM) (2).

### Microglial Are Myeloid Cells of the Brain Distinct From Peripheral Macrophages

Microglia are myeloid cells of the CNS parenchyma (3). With regard to ontogeny, microglia differ significantly from their macrophage relatives in other tissues. Both populations come from primitive macrophages, but at the germline level their paths of development diverge. Microglia come from yolk sac erythro-myeloid progenitors and settle the brain early in brain development before the blood-brain-barrier (BBB) closure (4). Migration of microglia progenitors to developing CNS is followed by rapid multiplication and creation of a pool of residual cells that are long-lived and have the ability to renew independently of the hematopoietic system. By the end of second postnatal week microglia become fully matured and express adult gene signature (5). Microglia share some genes with other mononuclear phagocytes, however, several transcripts are highly enriched in microglia, including CX3CR1, P2RY12,13, SOCS3, TREM2, TMEM119, GPR34, and SIGLEC (6). TMEM119, P2RY12, and SALL1 are considered microglia specific markers (5). When settled in the brain, peripheral macrophages possess intrinsic ability to express microglia genes, however the true identity of microglia is the function of both the ontogeny and environment (7). Environmental cues not only reassure microglial identity but also modulate enhancer landscapes in microglia. A recent study by Bennet et al. using a cell transplantation system in mice demonstrated environmental influence on microglial identity and the stunning plasticity of microglial cells (7), which has been further confirmed by Zhan et al. showing that murine microglia have the internal memory of their homeostatic signature, which allow returning to resting state (8). Despite significant changes in gene expression, which have been accompanied by morphological changes induced by *ex vivo* manipulations, after transplantation to the CNS microglia have quickly returned to their normal, homeostatic characteristics. Interestingly, hematopoietic stem cell—microglia like cells (HSC-MLC) that can, after transfer to CNS, imitate microglia, have been found to be enriched in genes associated with neurological diseases such as Alzheimer Disease (AD) (7). Microglia display a broad spectrum of phenotypes depending on environmental assemblage. Attempts to classify microglia as M1 or M2 like in case of other macrophages occurred far too simplistic and have failed, as evidenced by modern transcriptome profiling (9).

### Microglia Heterogeneity Is the Most Diverse During the Early Development

Microglia development process is highly dynamic and is characterized by changes in microglial states with unique sets of genes, morphology, distribution, and most likely function (10). Recent studies by Hammond et al. using single-cell RNA sequencing identified several microglial states present in mice brains throughout development, different ages, and conditions including injury (11). In this study, it has been found that the highest diversity of subpopulations of microglia with unique molecularity persist during early development. It has been proposed that unique gene patterns present during development represent specific transcriptional programs rather than the modulation of, already existing, one generic program. Interestingly, one population of microglia has displayed highly activated state despite the absence of any pathology and has been restricted to short postnatal period. Another identified microglial subpopulation has been present throughout life-span with increased prevalence in old age and injury with selective expression of the CCL4 chemokine. This population has also been enriched by expression of other inflammatory signals and has been proposed to be a specialized group to produce inflammatory responses. In general, in early development, microglial have been enriched in genes associated with metabolism, growth, motility, and proliferation and some of them became re-activated during injury and in aging brain. As the brain matures, microglia become less heterogeneous until aging and/or injury, which are characterized, as in development, by a large diversity and immature state.

A recent study by Li et al. has reported exceptional findings of spatiotemporal transcriptomic heterogeneity of microglia and other brain myeloid cells in six different brain areas and through three developmental stages in mice (12). Using deep single-cell RNA-seq technique, which enabled the detection of about three times as many genes per cell with higher detection rates as compared to previous scRNA reports, the study has demonstrated that regardless of the region of the brain, the adult microglia displayed vastly similar transcriptomes. This data has changed our perspective on microglial regional heterogeneity in adult brains based on previously reported findings. However, unlike adult cells, microglia in postnatal brain have been characterized by developmental complexity, with one subpopulation detected in the white matter unique in terms of morphological features such as round and ameboid shape. Interestingly, this newly identified white matter-associated microglia (WAM) have been found to be transiently present in the first postnatal week and be involved in phagocytosis of newborn oligodendrocytes and, most likely, astrocytes. It has been found that WAMs' activation is not dependent on TREM-signaling.

### In the Developing Brain Microglia Are Involved in Neurogenesis and Synaptic Pruning

During brain development, microglia development, and maturation is synchronized with neurogenesis. Neural and microglia cells exhibit extensive, dynamic physical interactions. The development and maintenance of microglia critically depends on the expression of Colony Stimulating Factor-1 Receptor (CSF-1R), which responds to its ligands CSF-1 and IL-34, released in the developing brain by neurons (13). Blockage of CSF-1R leads to microglia elimination and abnormal circuit connectivity in adult mice (14). Further microglia differentiation and maturation depend on TGF-β signaling. In a mouse model of limited CNS TGF-β expression, microglial maturation is altered (15). Microglia guide neuronal development mostly by processes like phagocytosis and factors production such as nerve growth factor (NGF) and tumor necrosis factor (TNF). The brain environment changes constantly during development, which elicits continual changes in microglial states. During late-embryonic and early-postnatal brain development, microglia are implicated in synaptic pruning, which eliminates excess and weaker synaptic connections. In the context of refinement of ipsilateral and contralateral retinogeniculate projections to the dorsolateral geniculate nucleus (dLGN) during the first postnatal week of life in mice, this process involves Complement Receptor 3 (CR3) (16). In this context, presynaptic elements destined for removal are decorated with “eat me” signals in the form of complement molecules C3 and C1q recognized by microglia, the only CNS cells with CR3 expression. Besides complement dependent pruning, mechanisms based on CX3CL1—CX3CR1 is also involved in synaptic elimination and maturation (17, 18). In mice, interference in refining neural circuits by eliminating complement-cascade or fractalkine-receptor signaling leads to circuit or connectivity abnormalities (16, 17). Some developmental processes based on complement dependent phagocytosis by microglia may occur in a gender-specific manner. This is the case in the process of refining dopaminergic circuity during adolescence in the nucleus accumbens. In male rats this process, based on the elimination of the dopamine receptors D1rs, is mediated via microglia C3-phagocytic activity (19). The mechanisms of dopamine receptor elimination in females remain to be determined.

### In the Adult Brain, Microglia Participate in Neuromodulation, Synaptic Plasticity, Learning and Memory Formation

In the adult brain, microglia perform many functions as diverse as neuronal support, synaptic modulation, reorganization of neuronal circuitry, and the production of significant amounts of antimicrobial peptides. Microglia communicate with neurons and neighboring cells via neurotransmitter receptors, purinoreceptors and ion channels. ATP is a key communicator of microglia with neurons and a key stimulator for microglial movement toward ATP sources. Using the larval zebrafish model Li et al. demonstrated a reciprocal cell-to-cell communication between microglia and neurons in a neuronal activity dependent manner based on purinergic receptor signaling (20). Activated neurons send “find me” signals (eg., ATP) through pannexin hemichannels to resting microglia. This is a signal for resting microglia to move processes toward targets, surround highly active neurons and consequently suppress neuronal activity.

It has been proposed that ATP signaling to purinergic receptors signals for release of Brain Derived Neurotrophic Factor (BDNF), which plays varied roles in neuronal differentiation, synaptic development, and plasticity. BDNF binds to neuronal TrkB, and regulates synaptic transmission and plasticity in mice, including formation of new synapses during learning (21). In one provocative experiment, deletion of *BDNF* from microglia did not change overall brain BDNF levels, but produced a phenotype showing deficits in a standard motor learning task accompanied by lack of new synapse formation in motor regions (21). In murine and human brain, microglial processes contact neuronal somata at specialized junctions regulated by purinergic signaling in microglia (22). These junctions are perturbed by neuronal injury and are coupled to neuronal mitochondrial activity (22).

An intriguing role is attributed to the neuron-microglia communication based on fractalkine/CX3CL1 produced by neurons and CX3CR1 expression limited to microglia (14). In neurogenesis and spatial learning, this interaction is particularly important because the lack of CX3CR1 reduces neurogenesis and lessens the efficiency of task learning (13). CX3CL1/CX3CR1 signaling has been characterized almost exclusively in mice, although there is an orthologous human chemokine-receptor pair.

### Microglia Rapidly Respond to Milieu Changes via Ion Channels, Cell Surface Receptors, and Epigenetic Reprogramming

The brain environment is highly dynamic, especially during development but also during adulthood and requires rapid responses from microglia. In a healthy adult brain in the optic tectum of larval zebrafish, microglia remain branched and scan the environment sensing neuronal activity and neurotransmitters reassuring neuronal proper functioning (20). Using two-photon imaging, a cluster of 100 genes called “sensome” has been discovered, which microglia use to detect changes in the environment. Two genes belonging to microglia sensome, a triggering receptor expression on myeloid cells 2 (TREM2) and CD33, are known risk factors for late-onset AD (14). Microglia sense changes in the environment using the processes that extend toward targets via differentially regulated non-directional as well as directed motility. Microglia processes monitor release of ATP, which is a main attractant and stimulus of microglia, entry of pathogens and fibrinogen, synaptic function, and activation of neurons. Microglia rapid and reversible responses to environmental changes are possible in part by activation of ion channels and cell surface receptors (23). Activation of ion channels leads to quick alteration of membrane potential that effects ramification, morphology, motility, surveillance, and other microglia functions. It has been found that microglia resting potential, ceaseless surveillance, and interleukin-1β release in murine models is dependent on the tonic activity of two-pore domain channel THIK-1, the main K+ channel, and that this process is independent on purinergic receptor P2Y12 activation (24). In contrast, the direct motility and extending processes toward tissue damage require activation of microglial P2Y12 receptors by ATP, and this process is independent on THIK-1 activity.

Dying neurons and non-functional synapses need fast clearance by microglia to prevent pathology. Microglia clearance phenotype is region specific and is tightly tuned by epigenetic mechanisms. It has been found that in mouse adult brain, striatal microglial phagocytic activity is epigenetically suppressed by the Polycomb repressive complex 2 (PRC2) as compared to the cerebellum (25). The latter requires higher clearance activity and the cerebellar microglia phenotype mimics that found during development and during certain neurodegenerative diseases. Of note, the cerebellum exhibits a spectrum of primary neurodegenerative processes, and recently has been implicated in the pathophysiology of AD although the neuropathological manifestations in cerebellum are less dramatic than those found in the forebrain (26, 27).

Epigenetic signals control microglial function also during the course of development and if altered might lead to neurodegenerative and psychiatric diseases. In mice, prenatal deletion of two class I histone deacetylases, Hdac1 and Hdac2, prominent regulators of epigenetic reprogramming and macrophage inflammatory responses, compromised microglial development. The deletion of these two genes in microglia in a mouse model of AD resulted in reduced amyloid deposition and improved cognitive function (28). It has been found in a mouse model of Alzheimer's disease that peripheral immune stimulation that induces acute immune training and tolerance in the brain might influence epigenetic reprogramming in microglia (29).

### Variants of Some Microglia Genes Are Risk Factors for Neurodegenerative Diseases

Microglia as part of the innate immunity respond to wide array of stimuli, including β-amyloid (Aβ), a toxic protein that accumulates in aging brains most likely as a consequence of slowing down Aβ metabolism and microglia phagocytic activity and is partially responsible for AD pathology. Microglia react to injury through morphological changes, increased proliferation, migration to the target, phagocytosis, activation of the NLRP3 inflammasome, and consequently the release of proinflammatory mediators (30). However, direct translations of cytokine functions that are well-defined in periphery may not operate in CNS context, with example of TNF-α displaying neuroprotective properties or TGF-β1 that is upregulated in aging and after CNS injury (6, 31).

All neurological diseases possess some inflammatory component and microglia are important contributors to brain pathology. Large-scale genome-wide association studies (GWAS) in AD model mice allowed for identification of more than 20 loci in immune-related genes associated with risk factors for neurodegenerative diseases with majority of them expressed by microglia or myeloid cells (31). One of the most intensely studied risk factors for neurodegenerative diseases is mutated TREM2, an innate immune receptor expressed by myeloid cells including microglia. During the early stages of brain development in mice, TREM2 plays a key role in elimination of extra synapses by regulation of microglia activity (32). TREM2 and Tyrobp (DAP12) form a signaling pair that suppresses inflammatory responses in mouse microglia *in vitro*, by reducing cytokine production and increases phagocytic activity that might lead to reduction of Aβ deposition and limitation of neurodegeneration. Several studies in mouse models for neurodegenerative diseases demonstrated opposing roles of TREM2 deficiency on Aβ and tau pathologies (two pathological hallmarks of AD) with amelioration of amyloid and exacerbation of tau pathology (33). For example, TREM2 deficiency in mice plays a stage-dependent role in contributing to amyloid deposition (34). TREM2 sustains metabolic fitness, energy homeostasis, proliferation, and survival in mouse microglia through mTOR signaling. TREM2 deficiency in a mouse model of AD causes metabolic and energetic imbalance followed by increased autophagy that resulted in a dysfunctional microglial state (35). R47H variant of TREM2 is one of the strongest single allele genetic risk factor for AD (36, 37). A mouse model of AD heterozygous for the TREM2 R47H allele showed loss of TREM2 function and enhanced neuritic dystrophy around plaques (38). These findings agree with other studies of mouse models and human subjects with R47H TREM2 mutations, consistently finding that microglia surround amyloid plaques, create a putative neuroprotective barrier, and limit plaque-associated neuritic dystrophy (39). This new role for “microglia barrier” in AD pathology has been reviewed (40).

During an injury or disease, microglia display a variety of phenotypes that can be detrimental or beneficial depending on the context (5). Human gene expression profiling obtained from frozen-post mortem AD specimens of superior frontal gyrus using RNA-Seq, has not been found to resemble any disease activation-related gene profile from animal models (41). Instead, this new profile of human Alzheimer's microglia/myeloid cells (HAM) resembled an “enhanced human aging” transcriptomic phenotype. The sole commonality between data obtained from animal models and HAMs involved genes associated with lipid metabolism and lysosomal biology. More data from human subjects are awaited since presently available animal models poorly reflect human pathophysiology. One of many problems concerns the usefulness of young mice with aggressive amyloid deposition phenotypes for studying age-related neurodegenerative diseases such as AD (42).

Cellular therapies with microglia serving as vehicles carrying genes or gene products to the CNS might be promising to confine neurological diseases. Recently, new approaches including usage of induced pluripotent stem cell (iPSC) microglia are potentially hopeful as therapeutic strategies (43). Microglia-like cells can be efficiently generated and enriched from multiple human embryonic stem and iPSC cells (44) although the *in vitro* context does not support expression of a transcriptome mirroring that seen in acutely isolated cells (45). Soluble cerebrospinal fluid TREM2 shows considerable promise as a biomarker for ongoing CNS pathology in AD (46). Interestingly, higher levels of CSF's TREM2 in comparison to phosphorylated tau is associated with attenuated cognitive decline in AD patients.

## Astrocytes

### Astrocytic Diversity Is Most Pronounced in Humans

Astrocytes (from Greek *astron* means *star*) have gained this name due to their characteristic star-like shape with long processes connecting with almost all types of CNS cells. They represent the largest group of glial cells with one astrocyte touching base with nearly 2 million synapses in the human brain. Unlike neurons, well-preserved among species, human astrocytes have undergone amazing changes during evolution (47), which most likely led to the development of unique human characteristics such as logical thinking and cognition. It has been found that human astrocytes extend 10 times more processes and are four times larger than mouse astrocytes. This correlates with the extreme expression of astrocytic PMP2, a fatty acid binding protein important for the normal structure of membrane lipids. Forced expression of PMP2 in the brains of neonatal mice resulted in an increase in diameter and number of astrocytes (47). Drawings by Ramon y Cajal at the beginning of the 20th century have shown a very complex structure of astrocytes, and current technologies confirm the extreme pleomorphism of astrocytes, especially in the human brain (48). The astrocytic phenotype is more defined by their mutual relations with neurons and the vascular system, then the expression of surface markers. Although glial fibrillary acidic protein (GFAP) is not completely astrocyte specific, for decades this marker has been used to identify astrocytes in the CNS (49). An attempt has been made to characterize astrocyte classes in adult murine CNS using dual staining of GFAP and a calcium-binding protein B (S100b) and nine astrocyte groups have been defined with the conclusion that the astrocytic phenotype is a function of the local microenvironment and operating requirements (50). Although they are highly heterogeneous, traditionally, astrocytes are divided into two major groups based on their location and structure. The first group includes protoplasmic astrocytes in the gray matter with “bushy” appearance and direct contact with blood vessels through their special anchorage at the end-foot. The second group contains fibrous astrocytes present in the white matter, contacting Ranvier nodes, and myelinated axonal pathways, where they support myelination (51).

### Astrocytic Ontogeny

Astrocytes are generated in the ventricular zone from the same progenitor cells as neurons and oligodendrocytes, called radial glial cells. Radial glial cells derive from neuroepithelial stem cells. In addition to generating the main classes of brain cells, they also serve as scaffold for localization of migrating neurons within developing brain layers (51). Astrogenesis, emergence of maturing astrocytes from radial glia, begins during mid-embryogenesis and continues postnatally (48). Locally astrocytes divide substantially throughout the first month of life.

### Astrocytes Contribute to Formation of Neural Circuits

Synaptogenesis takes place across approximately the same stages of development as does astrogenesis, beginning before many astrocytes are present and continuing postnatally in the presence of increasingly-numerous astrocytes (52). Synapses are interconnecting elements between two neurons that allow the transmission of signals in neuronal networks. It takes commitment from both, the glial cells and neurons to create a functional synapse in which immature neurons guided by astrocytes find partners to make connections (53). The recognition of astrocytes in the formation of synapses and neural circuits have come from experiments with neuronal cell culture showing the inability of isolated neurons to survive and form synapses without the addition of astrocytes or factors that they secrete (54). The astrocytic modulation of synaptogenesis is mediated by contact between cells and secreted factors. Cell-cell contact is particularly important for embryonic neurons to form excitatory and inhibitory synapses and is partially facilitated by cell adhesion molecules present on both parties: astrocytes and neurons. For example, astrocytes express neuroligin that binds to neuronal neurexin, which is important not only for synaptic contact, but also for astrocyte morphology and accurate synaptic function in the mouse cortex (55). Additionally, astrocytes influence the growth and development of synapses by secreting stimulatory and inhibitory mediators associated with synaptogenesis. Astrocytes inhibit synaptogenesis by producing two negative regulators: Brain Derived Neurotrophic Factor (BDNF) and Secreted Protein Acidic, Rich in Cysteine (SPARC). SPARC limits the levels of AMPA receptors (postsynaptic glutamate receptors, whose activation leads to strengthening of the synapse), ultimately modulating the activity-dependent elimination of synapses in mice (56). SPARC antagonizes presynaptic Hevin/SPARCL1, which together with Thrombospondins (TSP1,2) plays an important role in the formation of glutamatergic synapses and provides synaptic stabilization and consolidation. Formation of functionally active synapses is maintained by heparan sulfate proteoglycans, glypican 4 and 6 (Gpc4,6) in mouse models (57). Gpc4 secreted by murine astrocytes acts on presynaptic accumulation of neuronal pentraxin 1 (NP1), which further stimulates active synapse formation by clustering of AMPA receptors rich in GluA1 (58). The increase in AMPA receptors and the reduction of gamma-aminobutyric acid (GABA) receptors present in inhibitory synapses is regulated by astrocytic TNF-α. The presynaptic activity and upregulation of synaptic transmission is partially maintained by cholesterol, lipid synthesized by astrocytes, which in combination with ApoE is transported to neurons. Mice with astrocytes with interrupted lipid synthesis show impaired synaptic development and plasticity (59).

During synaptogenesis but also in adult mouse brain, synapses are removed in activity-dependent fashion to refine neural circuits. This task is allocated to glia, including microglia and astrocytes (60). In mice, astrocytes utilize Megf10 and Mertk to target synapses for removal by direct engulfment. Astrocytes also contribute to refining neural networks through production of soluble factors. As one example, astrocytes release TGF-β, which increases complement C1q expression in neurons and makes them visible for phagocytosis by microglia (53). Synapse engulfment is also regulated by astrocytic IL-33, a member of the IL-1 family, via IL1RL1 receptor on phagocytic murine microglia (61). Interestingly, microglial TREM2, implicated in risk for neurodegenerative diseases, is required for microglia to signal to astrocytes to limit their synapse uptake. Mice lacking TREM2 show reduced synapses resulting from loss of this regulatory mechanism during development. In TREM2-null mice, high-fat diet during adulthood reignites astrocytic synapse removal (62), showing that astrocyte engulfment of synapses is under active restraint in adult mice.

### Astrocytes Guard the Proper Functioning of Synaptic Circuits

In the adult brain, astrocytes continue to guard proper functioning of the brain and neurons. Astrocyte processes are an inseparable part of synapses, and are well-positioned to respond and/or control the concentration of neurotransmitters via specific membrane receptors and/or their uptake by membrane transporters like AMPA and N-methyl-D-aspartate (NMDA) glutamate transporters (63). Glutamate, a major neurotransmitter released by neurons is toxic in excess and its proper synaptic concentration is maintained by astrocytes. Astrocytes take in glutamate, convert it to glutamine and in this form shuttle it back to neurons. Glutamine acts as a precursor for glutamate and GABA. Uptake of glutamate by neurons is partly facilitated by fractalkine, a chemokine produced by neurons that promotes neuroprotection, and this action requires astrocyte-microglia communication, because only microglia express the receptor for fractalkine in CNS. Astrocytes have receptors for neuronal mediators, including G-protein-coupled receptors associated with intracellular calcium Ca signaling (53). Astrocyte activity can be visualized by imaging changes in intracellular Ca2+ levels and it is widely accepted that the dynamic communication between astrocytes and neurons studied in murine models is maintained by purinergic receptors and is fortified by the calcium waves and oscillations (49). This type of signaling is used by astrocytes to control many vital function, such as neuronal synchronization, trophic factors concentration and neurotransmitter uptake, modulation of K+ uptake, vascular size sensing and gene expression, and most likely expression of disease-related molecules (63). Reducing astrocyte calcium signaling in mouse striatum confirmed its functional significance (64). In particular, mice demonstrated a marked phenotype of increased repetitive self-grooming associated with increased GABAergic signaling to astrocytes, and mediated by striatal medium spiny neurons (64).

Elevation in astrocyte calcium levels affects production and release of neuromodulators called gliotransmitters, such as ATP, GABA, glutamate, d-serine, lactate, and TNF-α, which affect the plasticity of neurons and their communication with microglia and endothelial cells (49). TNF-α, at physiological levels and produced predominantly by microglia, is needed for astrocytic glutamate release. However, microglial TNF-α at high concentrations causes excitotoxic effects by suppressing astrocyte uptake of glutamate (65).

### Astrocytes Display Wide Array of Homeostatic Functions

Astrocytes are interconnecting units with end feet contacting elements of the BBB, which provide nutrients and oxygen. Astrocytes also interact physically with neurons, which rely on this supply. Proper function of blood-and cerebral fluid-brain barriers are supervised by astrocytes. Astrocytes regulate BBB function in part by secretion of factors that modulate barrier properties in context-dependent fashion (66). The structural components of astrocytic endfeet also mediate interactions with the BBB. In particular, astrocyte endfeet are typified by orthogonal array particles, which contain the widely-expressed potassium channel, Kir4.1, and astrocyte-restricted aquaporin-4 water channels. These components support BBB functions of controlling brain potassium ion and water balance. The levels of reactive oxygen species within CNS are also under astrocytic supervision. Astrocytes and neurons build a strong metabolic connection. Astrocytes are major sources of brain cholesterol, crucial for the composition of neural membranes, and a precursor for signaling molecules. In addition, glucose stored exclusively in astrocytes in the form of glycogen allows the use of lactate as a source of energy not only for neurons, but also for other brain cells (53).

### Astrocytes Respond to Insult by Upregulation of GFAP and Hypertrophy

The main role of astrocytes in the brain is to protect from damage to the CNS and to repair the nervous tissue after the injury, so it is not surprising that astrocytes are involved in wide array of neurological disorders. The response of astrocytes in neurological disorders such as trauma, neuroinflammation, and neurodegeneration as a physiological defense response is called astrogliosis. Activated astrocytes are characterized by a different molecular pattern, morphology, and function as compared to their normal counterparts. Extensive GFAP expression is a hallmark of reactive astrocytes. Normal astrogliosis after brain injury is associated with inositol 1,4,5-triphosphate (IP3)-dependent signaling pathway and N-cadherin upregulation (67). Reactive astrocytes are essential for scar formation, inhibition of the spread of inflammatory cells, and repair of blood-brain barrier insults. Recent findings show that scar formation may stimulate axonal regrowth after severe spinal cord injury in adult mice (68). In addition, during astrogliosis after invasive injury in mice, the formation of new neurons and oligodendrocytes from stem-like reactive astrocytes has been observed (69). In a healthy brain, astrocytes are organized in non-overlapping domains that can play a role in neuropathology. Reactive astrocytes lost their domain organization in experimental models of epilepsy, but are preserved in the animal model of AD. So far the significance of astrocytic domains in health and disease remains unclear. The effect of reactive astrogliosis in disease is complex: reactive astrocytes can be both beneficial and harmful to surrounding cells and can solve or worsen initial CNS damage. This process has a favorable outcome during acute stress or focal cerebral ischemia, but can limit regeneration at a later stage. Reactive astrocytes may be neurotoxic when producing reactive oxygen species or certain inflammatory cytokines. Local elimination of activated astrocytes improved axonal regeneration after injury in postnatal mice (70). Many chronic neurological disorders are accompanied by chronically stressed, degenerated, and atrophic astrocytes with loss of function, which adds to the progression of the disease. Reactive astrogliosis is a complicated phenomenon, however, it is common in various CNS pathologies. Molecular changes in astrocytes are highly context specific. Although there is a set of genes that are consistently upregulated in various pathologies, about 50% of altered gene expression varies depending on the type of brain damage (67). Unfortunately, at this point, the lack of specific markers for heterogeneous, region-specific astrocyte subtypes significantly limit our understanding of the functional consequence of reactive gliosis in different neurological diseases. In addition, the disadvantage in astrocyte, but also microglial research, is the use of rodent models and *in vitro* settings that poorly reflect conditions prevailing in the human CNS.

## Astrocyte-Microglia Communication

**Figure 1:**
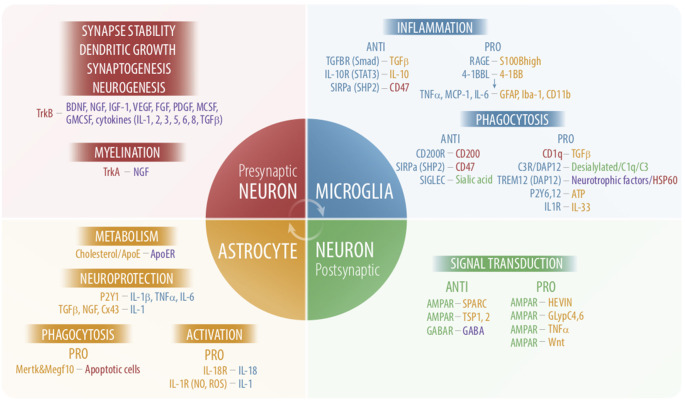
Schematic overview of some interactions among astrocytes, microglia and neurons. Molecules participating in cross-talk and their cellular sources are shown in the same colors. Functions are results of these interactions are depicted in black capital letters next to the cell types where the particular process take place. Purple color reflects multiple sources. AMPAR, α-amino-3-hydroxy-5-methyl-4-isoxazolepropionic acid receptors; APOE, apolipoprotein E; BDNF, brain derived neurotrophic factor; DAP12, DNAX activation protein of 12 kDa; FGF, fibroblast growth factor; GABA, γ-aminobutyric acid; GFAP, glial fibrillary acidic protein; GMCSF, granulocyte macrophage colony stimulating factor; lba-1, ionized calcium binding adaptor protein 1; IGF-1, insulin- like growth factor-1; MCSF, macrophage colony-stimulating factor; NGF, nerve growth factor; PDGF, platelet-derived growth factor; RAGE, advanced glycation end products; SIGLEC, sialic acid-binding immunoglobulin-type lectins; SIRPa, signal regulatory protein; SHP2, SH2-domain-containing protein tyrosine phosphatase 2; SPARC, secreted protein, acidic and rich in Cysteine; TREM, triggering receptors expressed on myeloid Cells; Trk, neurotrophin receptor tyrosine kinase; TSP1,2, Thrombospondin1,2; VEGF, vascular endothelial growth factor.

Astrocytes are distributed in a complex network that is connected by gap junctions and are found in all operational areas of the brain and spinal cord and all neuronal layers, and thus bridge and influence neural circuits that are not directly connected. In addition, astrocytes form long processes with the end feet structures that allow communication with blood vessels, another dense multicellular network. Microglia, as revealed by live imaging, are restless cells and constantly move their processes through the brain environment (71, 72). Astrocytes-microglia together with glutamatergic neurons constitute a unit called the “quad-partite synapse,” which is necessary for the operation of the circuit and is based on neuro-immune communication (73). Some interactions between astrocytes and microglia in the neuronal context are depicted in Figure 1.

### Different Ways of Communication Typify Astrocyte-Microglial Cross-Talk

Cross-talk between astrocytes and microglia is maintained in part via secreted mediators, such as growth factors, neurotransmitters and gliotransmitters, cytokines, chemokines, innate-immunity mediators and tissue damage molecules (e.g., ATP), mitogenic factors, NO, ROS, and metabolic mediators such as glutamate, that can be used for cell metabolism and may also mediate tissue changes. In addition, astrocytes, microglia, and neurons communicate via releasing and responding to extracellular vesicles. Extracellular vesicles function over long distances and can contain active biomolecules, including mRNA and miRNA, that are capable of modulating gene expression in distant cells. A proteomic study showed that the extracellular vesicle derived from *in vitro* ATP stimulated microglia were able to induce a molecular reaction in targeted astrocytes (74). Of note, extracellular vesicles may participate in pathogenesis of neurodegenerative disorders by transporting and transferring toxic aggregates, such as tau and Aβ (75). Reduced levels of presynaptic proteins in exosomes derived from neurons have been reported early in disease, and their quantification in patient plasma may carry prognostic and therapeutic value in neurodegenerative diseases (76).

Another route for astrocyte-microglia was described in mice: cross talk may proceed through the gut–brain axis by which metabolites of dietary tryptophan controlled by commensal flora act directly on CNS-resident microglia and their production of Vascular Endothelial Growth Factor-β (VEGF-β) and TGFα, which regulate astrocyte pathogenic activities during inflammation and neurodegeneration (77).

Purinergic signaling though P2Y receptors, expressed in astrocytes and microglia may play a major role in the communication of microglia with astrocytes during the inflammatory response. For example, ATP derived from astrocytes, which binds P2Y12 and P2Y6 expressed by microglia, promotes microglial phagocytosis, and processes extension in rats (78). Binding of ATP by microglia and astrocytes, contingent on which purinergic receptor is expressed, may evoke calcium currents in both cell types, and the production of inflammatory cytokines by cultured dorsal horn microglia (79).

### Cytokines Are Important Mediators Between Astrocytes and Microglia

Reactive glia including astrocytes and microglia can express and secrete canonical cytokines such as IL-1β, IL-6, TNF-α, IL-18, TGF-β, and IL-10 after acute tissue injury (80). Contingent on receptor expression, these cytokines function in both autocrine and paracrine manner. They are differentially produced by microglia, astrocytes, oligodendrocyte progenitor/NG2+ cells, and neurons in context-dependent fashion, being expressed when cells sense dyshomeostasis. In the neurotypical context, these cytokines occasionally also play a key roles in physiological processes (81). For example, the IL-33 cytokine of the IL-1 family expressed in developing astrocytes in the spinal cord and thalamus plays a role in synaptic refinement, signaling to microglial IL-1RL1. In gene-targeted mice, IL-33 deficiency results in a surplus of excitatory synapses and a hyper-excitable intrathalamic circuit (61).

In the context of brain injury in mice, cytokines, such as IL-1β, TNF-α, and IL-6 released by microglia, regulate astrocytic responses, and lower astrocyte P2Y1 receptor to enable tissue remodeling and repair (82). By constrast, another set of cytokines produced by activated mouse microglia *in vitro* and composed of IL-1α, TNF-α and complement factor Cq1, induces in mouse astrocytes a putative neurotoxic state astrocytes “A1” (83). Investigation of astrocytes in Huntington Disease (HD) cingulate gyrus using snRNA-Seq, with extensive confirmatory steps for RNA and protein expression, and comprehensive informatics, disclosed three astrocytic states that mapped to transcriptomic clusters (84). This study disclosed no evidence in favor of A1 (neurotoxic) or A2 (neuroprotective) astrocytic states in human neurodegenerative disease.

TGF-β and IL-10 are antagonists to some TNF-α and IL-1 activities and thus participate in regulating the inflammatory response. TGF-β produced by astrocytes signals to microglia among other cells, decreasing expression of some inflammatory mediators. Microglial TGF-β reduces subacute neuroinflammation after stroke in mice (85). Cytokine-activated astrocytes can promote neurogenesis in adult mice in the sub-ventricular zone (86).

### Cross-Talk in Disease

As a result of their diverse and complex roles, microglia and astrocytes contribute critically to brain homeostasis, and are now accepted as important disease modifiers.

In the context of inflammatory neurological diseases, cross-talk between astrocytes and microglia seems particularly important. Both microglia and astrocytes are considered to be part of the innate immune system based on their ability to produce immunomodulators and expression of receptors associated with innate immunity, such as complement receptors or Toll-like Receptors (TLRs). For example, the response to LPS requires TLR-4, which is present on innate immune cells and microglia. Although murine *Tlr4* is expressed only in microglia, microglia and astrocytes acutely isolated from human brain both express *TLR4*.

Appropriate astrocyte-microglia cross-talk in disease is necessary for astrocytes to support neuronal survival and function after acute injury. Modeling in mice suggests that microglia constitute a first line of defense, demonstrating activation, and fast recruitment to sites of damage to phagocytose dead cells and debris (87). Secondary to microglial reaction is the activation of astrocytes, which release inflammatory mediators that signal to microglia and can recruit MIG infiltrating hematogenous cells including monocyte-derived macrophages. Reactive astrocytes upregulate GFAP and undergo morphological changes leading to the formation of glial scars, which may limit damage within the affected area (88).

### Response in Disease Is Context Dependent

Whether glial cells adopt a phenotype that aggravates tissue injury or promotes brain repair, most likely depends on a basic set of factors, such as the nature of the damaging element, severity and time course of injury, and precise constellation of signals from the environment. The response largely depends on the disease context.

In obesity-induced hypothalamic inflammation of mice, the responses are induced by inflammatory cytokines such as TNF-α, CCL2, and IL-6, and involve canonical gliosis markers (GFAP, Iba-1, and CD11b). This reaction results from direct binding of astrocytic 4-1BB, a member of TNFRSF to its ligand 4-1BBL expressed on microglia. This direct conversation between glia cells promoted monocyte/macrophage proliferation and migration (89). A three-party cross-talk among microglia, astrocytes, and neurons has been identified in the study of viral infection of CNS of mice, via the olfactory route. Protection against the further spread of viral infection has been maintained by an early innate barrier composed mainly of microglia, whose response was regulated by strong IFNAR signaling from neurons and weaker signaling from astrocytes (90).

In summary, glial cells regulate and control each other's function, migration and reactions. A noticeable bi-directional conversation between astrocytes and microglia is evident in the context of neurological disorders. The astrocytes-microglia interplay may determine the phenotype that astrocytes and microglia express during disease. Current therapies for the treatment of neurological disorders and clinical trials based on blocking inflammatory reactions are manifestly insufficient. It is useful to maintain awareness that the CNS environment implicates astrocytes and microglia in programs and functions that cannot be understood in the context of typical immunological reactions. The languages of microglia and astrocytes will be the key to understanding this complex system composed of cells of unimaginable diversity and plasticity, and of manifold yet-unknown functions.

## Conclusions and Future Prospects

We find ourselves in an unprecedented era of progress in understanding neuroscience and, along with it, glial biology. These insights extend seamlessly from development through aging and disease, which can now be regarded as experiments of nature. The drivers of this progress extend from optogenetics and novel imaging techniques through germline genetics, multi-omics and bioinformatics, through innovative cell and organoid models. The methods are deployed by an increasingly diverse, committed and interactive community of researchers, many of whom arrived from other disciplines as far afield as mathematics, physics, and immunology. Comprehension of the molecular interactions among cells in tissues and organs will lay the biological foundation to identify the right drug targets and modalities for successful assault on disorders of the developing, injured, infected, or aging nervous system.

## Author Contributions

AM wrote the first draft, performed data mining, generated the figure, and contributed to editing. RR conceived the topic and outline of the review, recruited AM to participate, supervised the preparation of the drafts and figure, and performed final edits. All authors contributed to the article and approved the submitted version.

## Conflict of Interest

RR is employed by the company Third Rock Ventures. The remaining author declares that the work was conducted in the absence of any commercial or financial relationships that could be construed as a potential conflict of interest.
